# Supplementary figures and images for: Genes involved in immune, gene translation and chromatin organization pathways associated with Mycoplasma ovipneumoniae presence in nasal secretions of domestic sheep
Source: PLoS One. 2021 Jul 12;16(7):e0247209. doi: 10.1371/journal.pone.0247209 (PMC8274911; doi:10.1371/journal.pone.0247209)

## Slide 1
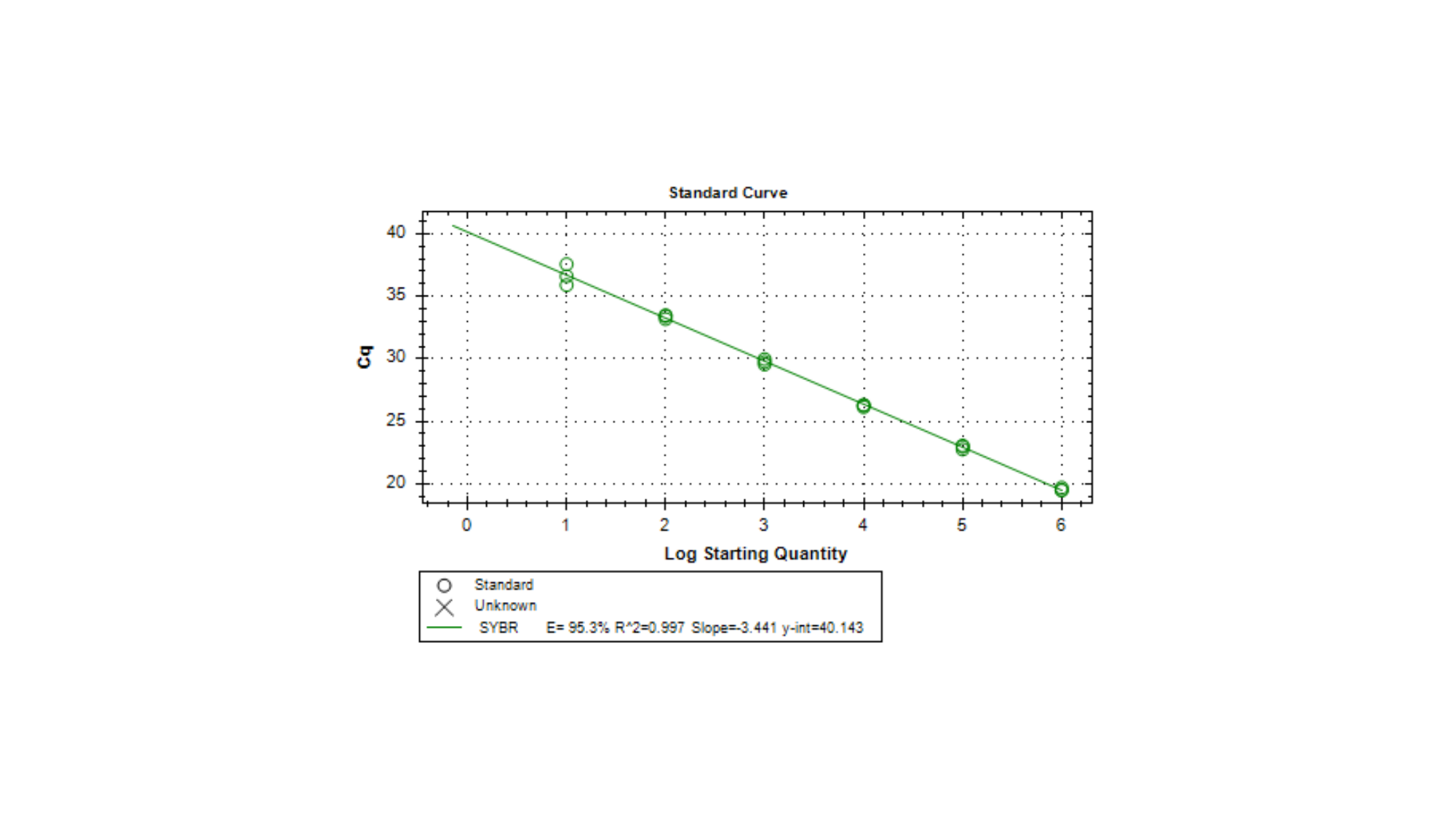

Supplement: S1 Fig — (PPTX) [file pone.0247209.s001.pptx]
