# Supplementary material for: Genes involved in immune, gene translation and chromatin organization pathways associated with Mycoplasma ovipneumoniae presence in nasal secretions of domestic sheep
Source: PLoS One. 2021 Jul 12;16(7):e0247209. doi: 10.1371/journal.pone.0247209 (PMC8274911; doi:10.1371/journal.pone.0247209)
Supplement: S1 Table — (DOCX) [file pone.0247209.s002.docx]

Supplemental Table 1. Name of significant SNPs on OvineHD Beadchip, rs number, the animal set in which the SNPs were identified, the minor allele, and trend of *M. ovipneumoniae* transformed log_10_ mean DNA copy number associated with minor allele.

| Illumina SNP name | dbSNP rs# cluster id | Animal Set | Minor Allele | Trend |
| --- | --- | --- | --- | --- |
| oar3_OAR6_101266504 | rs159990985 | All | C | Higher |
| oar3_OAR6_101279171 | rs159991022 | All | T | Higher |
| oar3_OAR9_13890848 | rs160633679 | Rambouillet | A | Higher |
| oar3_OAR17_53882329 | rs160856157 | Polypay | C | Higher |
| oar3_OAR4_8831863 | rs398278706 | Rambouillet | A | Higher |
| oar3_OAR7_76225469 | rs400632990 | All | T | Higher |
| oar3_OAR7_91908439 | rs402243563 | Polypay | A | Higher |
| oar3_OAR17_53261568 | rs402253048 | Polypay | G | Higher |
| oar3_OAR3_108633720 | rs403152750 | Rambouillet | A | Higher |
| oar3_OAR3_108638337 | rs404662185 | Rambouillet | G | Higher |
| oar3_OAR15_19011353 | rs406524055 | Suffolk | T | Higher |
| OAR15_15503325.1 | rs406526355 | Suffolk | C | Higher |
| oar3_OAR15_15453723 | rs406613692 | Suffolk | T | Higher |
| oar3_OAR3_108714118 | rs407012017 | Rambouillet | C | Higher |
| oar3_OAR3_108640566 | rs409357561 | Rambouillet | G | Higher |
| oar3_OAR15_18879871 | rs409884491 | Suffolk | A | Higher |
| oar3_OAR15_15371024 | rs410915115 | Suffolk | T | Higher |
| oar3_OAR7_76212720 | rs411153659 | All | A | Higher |
| oar3_OAR15_15401315 | rs413355105 | Suffolk | T | Higher |
| oar3_OAR22_10938408 | rs413436283 | Rambouillet | A | Higher |
| oar3_OAR15_17220107 | rs418601541 | Suffolk | G | Higher |
| oar3_OAR7_53135906 | rs420722286 | Suffolk | G | Higher |
| oar3_OAR7_91281519 | rs424565925 | All | T | Higher |
| oar3_OAR7_76205432 | rs426816169 | All | T | Higher |
| oar3_OAR7_76226838 | rs427671730 | All | G | Higher |
| oar3_OAR10_67992867 | rs429475222 | Rambouillet | G | Higher |
| oar3_OAR22_10929142 | rs429858348 | Rambouillet | T | Higher |
| oar3_OAR15_19011908 | rs590224797 | Suffolk | T | Higher |
